# Cellular and transcriptional impacts of Janus kinase and/or IFN-gamma inhibition in a mouse model of primary hemophagocytic lymphohistiocytosis

**DOI:** 10.3389/fimmu.2023.1137037

**Published:** 2023-04-27

**Authors:** Sabrin Albeituni, Ninad Oak, Heather S. Tillman, Alexa Stroh, Camille Keenan, Mackenzie Bloom, Kim E. Nichols

**Affiliations:** ^1^ Department of Oncology, St. Jude Children’s Research Hospital, Memphis, TN, United States; ^2^ Department of Pathology, St. Jude Children’s Research Hospital, Memphis, TN, United States

**Keywords:** hemophagocytic lymphohistiocytosis (HLH), cytokines, inflammation, interferon-gamma (IFNg), Janus kinase (JAK), ruxolitinib, emapalumab

## Abstract

**Background:**

Primary hemophagocytic lymphohistiocytosis (pHLH) is an inherited inflammatory syndrome driven by the exuberant activation of interferon-gamma (IFNg)-producing CD8 T cells. Towards this end, ruxolitinib treatment or IFNg neutralization (aIFNg) lessens immunopathology in a model of pHLH in which perforin-deficient mice (*Prf1*–/–) are infected with Lymphocytic Choriomeningitis virus (LCMV). However, neither agent completely eradicates inflammation. Two studies combining ruxolitinib with aIFNg report conflicting results with one demonstrating improvement and the other worsening of disease manifestations. As these studies used differing doses of drugs and varying LCMV strains, it remained unclear whether combination therapy is safe and effective.

**Methods:**

We previously showed that a ruxolitinib dose of 90 mg/kg lessens inflammation in *Prf1*–/– mice infected with LCMV-Armstrong. To determine whether this dose controls inflammation induced by a different LCMV strain, we administered ruxolitinib at 90mg/kg to *Prf1*–/– mice infected with LCMV-WE. To elucidate the impacts of single agent versus combination therapy, *Prf1*–/– animals were infected with LCMV, treated or not with ruxolitinib, aIFNg or both agents, and analyzed for disease features and the transcriptional impacts of therapy within purified CD8 T cells.

**Results:**

Ruxolitinib is well-tolerated and controls disease regardless of the viral strain used. aIFNg, administered alone or with ruxolitinib, is most effective at reversing anemia and reducing serum IFNg levels. In contrast, ruxolitinib appears better than aIFNg, and equally or more effective than combination therapy, at lessening immune cell expansion and cytokine production. Each treatment targets distinct gene expression pathways with aIFNg downregulating IFNg, IFNa, and IL-6-STAT3 pathways, and ruxolitinib downregulating IL-6-STAT3, glycolysis, and reactive oxygen species pathways. Unexpectedly, combination therapy is associated with upregulation of genes driving cell survival and proliferation.

**Conclusions:**

Ruxolitinib is tolerated and curtails inflammation regardless of the inciting viral strain and whether it is given alone or in combination with aIFNg. When administered at the doses used in this study, the combination of ruxolitinb and aIFNg appears no better than treatment with either drug alone in lessening inflammation. Further studies are warranted to elucidate the optimal doses, schedules, and combinations of these agents for the treatment of patients with pHLH.

## 1 Introduction

Cytokine storm syndromes (CSS) are characterized by the excessive production of proinflammatory cytokines due to dysregulation of immune responses. Failure to recognize and properly manage these exacerbated immune responses can lead to multiorgan failure and death. Primary hemophagocytic lymphohistiocytosis (pHLH) is one such CSS that is due to germline pathogenic variants affecting genes required for lymphocyte cytotoxic function. This cytotoxic function is critical for normal immunoregulation, as shown following Lymphocytic Choriomeningitis virus (LCMV) infection of perforin-deficient (*Prf1*−/−) mice, which develop fatal immunopathology due to heightened CD8 T cell expansion and cytokine production. In this mouse model, the cytokine interferon gamma (IFNg) is an important driver of disease pathophysiology (1, 2) with antibody-mediated neutralization of IFNg (aIFNg) ameliorating disease and significantly prolonging survival (2). It was recently reported that the IFNg neutralizing antibody emapulumab (Gamifant^®^), administered with dexamethasone, improves the clinical manifestations and overall survival of children with pHLH (3). Based on these findings, emapalumab was granted approval by the Food and Drug Administration for the treatment of children and adults with refractory or recurrent pHLH or intolerance of conventional HLH therapies.

Many of the cytokines that are elevated in pHLH, including IFNg, and interleukin (IL)-2, IL-6, granulocyte macrophage colony stimulation factor (GM-CSF), and IL-10, signal through the Janus Kinases (JAK) and Signal Transducers and Activators of Transcription (STAT) pathway (4). Building on this information, several pre-clinical mouse studies as well as human case reports and clinical trials have demonstrated that the JAK1/2 inhibitor, ruxolitinib, is effective at lessening inflammation and curtailing disease manifestations (5–9).

Despite their beneficial effects, neither aIFNg nor ruxolitinib completely abrogate the signs of disease. In this regard, a recent study combining treatment with “high” doses of ruxolitinib (90 mg/kg twice daily) and aIFNg antibody (40 mg/kg; roughly 1mg, every 3-4 days) revealed toxicity and decreased survival of LCMV WE-infected *Prf1*−/− animals (10). In contrast, a separate study incorporating “low” doses of aIFNg antibody (200 μg every 3 days) and ruxolitinib (4 mg/kg twice daily) reported superior suppression of inflammation in LCMV Armstrong-infected *Prf1*−/− mice (11). To date, it has remained unclear whether the opposing outcomes in these studies were due to differences in the viral strains used (with LCMV Armstrong being more neurotropic and LCMV WE more hepatotropic (12)) or rather, to differences in the doses of aIFNg and/or ruxolitinib administered. To address this question, we first tested the tolerability and efficacy of “high” dose ruxolitinib in LCMV WE infected *Prf1*−/− animals and found that the drug was well-tolerated and lessened inflammation. To further determine the impacts of combination therapy, we administered high dose aIFNg, ruxolitinib, and the combination of aIFNg and ruxolitinib to LCMV-Armstrong infected *Prf1*−/− animals. Consistent with our prior report (7), aIFNg and ruxolitinib differentially impacted disease manifestations with aIFNg reversing anemia, and ruxolitinib reducing immune cell expansion and cytokine production. Notably, combined treatment with aIFNg and ruxolitinib was no better than therapy with either agent alone in ameliorating each of these disease parameters. Transcriptional profiling of splenic CD8 T cells revealed that aIFNg and combination treatment targeted IFNg response genes more effectively than ruxolitinib; however, combination treatment paradoxically induced the expression of genes involved in IL-2 and STAT5 signaling pathways, as well as E2F and MYC targets. As cytokines function in networks that counter regulate one another, these studies suggest that combining cytokine-targeting agents may not always function in an additive fashion to dampen inflammation. Rather, the doses and schedules of these medications must be carefully titrated to confer maximum benefit while minimizing pro-inflammatory or other unanticipated effects.

## 2 Materials and methods

### 2.1 Mice

Perforin knockout (*Prf1*−/−) mice (C57BL/6 Prftm1Sdz/J) were purchased from The Jackson Laboratory. Sex and age matched mice between 6-12 weeks of age were used for these studies. Mice were housed in specific pathogen-free facilities at St. Jude Children’s Research Hospital. All experimental protocols were approved by the Institutional Animal Care and Use Committee and the Institutional Biosafety Committee.

### 2.2 Primary HLH model and administration of aIFNg and/or ruxolitinib

*Prf1*−/− mice were infected intraperitoneally (i.p.) with 200 plaque-forming units (PFU) LCMV strain WE (c2.2) (provided by Juan Carlos de la Torre, Scripps Research Institute, La Jolla, CA) or 2×10^5^ PFU of LCMV Armstrong (provided by John Wherry, University of Pennsylvania, Philadelphia, PA). For mice infected with LCMV WE, ruxolitinib (provided by Ross Levine, Memorial Sloan Kettering Cancer Center, New York, NY; dissolved in citrate buffer [0.1M, pH 3.5] with captisol [20% w/v]) was administered by oral gavage twice daily at 90 mg/kg, from days 7 to 20 post infection (p.i.; LCMV WE) or from days 4 to 8 p.i. (LCMV Armstrong). These schedules of ruxolitinib administration were chosen based on prior reports testing the therapeutic effects of this drug in *Prf1*−/− mice infected with LCMV WE (6) or LCMV Armstrong (7, 8, 13). In each of these models, mice were showing early signs of inflammation at the time of drug initiation [ (6, 10, 13) and our unpublished data]. Anti-IFNg neutralizing antibody (clone XMG1.2; BioXCell) was administered i.p. at 500 μg per mouse on days 4 and 7 p.i. (LCMV Armstrong). In the current study, we administered aIFNg at 500 μg per mouse instead of 40 mg/kg (which equates to ~1mg/mouse). We chose 500 μg based on our prior observation that IFNg was equally neutralized in mice receiving 500 μg or 1mg of aIFNg. For example, both doses conferred similar improvements in hematologic and cellular phenotypes in LCMV-Armstrong infected *Prf1*−/− mice, as well as comparable inhibition of STAT1 phosphorylation in blood monocytes (7). Mice were euthanized on day 21 p.i. (LCMV WE) or 9 p.i. (LCMV Armstrong) and examined for HLH manifestations. Over the course of LCMV WE infection, clinical scores were determined and calculated in a blinded fashion as described (14). Briefly, mice were scored based on weight loss (0–3), stance (0–3), skin tenting (0–2), coordination (0–3), conjunctivitis (0–2), and ascites (0–2). Mice that lost more than 20% of their body weight or had a score of over 11 were considered moribund and were euthanized.

### 2.3 Complete blood counts

Heparinized blood was collected by cardiac puncture and CBCs analyzed using a Forecyte multi-species hematology system (Oxford Science).

### 2.4 Serum cytokines

The concentration of serum cytokines was measured using a Milliplex Map Mouse Cytokine/Chemokine Magnetic Bead Panel (EMD Millipore) per manufacturer’s instructions. Results were collected using BIO-Plex 200 System (Bio-Rad) and analyzed using xPONENT software. CXCL9 was measured with Quantikine ELISA and soluble sCD25 was measured with ELISA kit (R&D Systems) as per manufacturer’s instructions. Results were collected and analyzed using Hidex Sense microplate reader.

### 2.5 Liver histology

Liver sections were fixed in 10% neutral buffered formalin before standard histological processing, sectioning, and staining with hematoxylin and eosin (Richard-Allan Scientific). Several sections of the evaluated tissues were placed together on the same slide to provide representative regions of the entire organ for the histopathology analyses. Slides were then evaluated with a Nikon Eclipse Ni microscope and then digitized to scalable images up to a 20x objective lens with an Aperio ScanScope scanner (Leica Biosystems). Normal tissue, inflammation and clear space/glass were segmented, and the percentage of tissue area infiltrated by immune cells was quantified as well as the number of inflammatory foci on 2x magnification static images of the tissues using the FIJI image analysis program. Histological analysis was performed in a blinded fashion.

### 2.6 Flow cytometry and cell sorting

Spleens were manually homogenized and lysed with Ammonium-Chloride-Potassium (ACK) buffer. Aliquots of single-cell suspensions were resuspended in fluorescence-activated cell sorting buffer (FACS) buffer containing 1% bovine serum albumin (BSA) and 0.05% sodium azide. Cells were then stained with fluorescently labeled antibodies for 30 minutes at 4°C. The following fluorescently labeled antibodies were used for surface staining: TCRb (H57-597), F4/80 (BM8.1), NK1.1 (PK136), Ly6C (HK1.4), CD11c (N418), CD11b (M1/70), CD8 (53-6.7), CD19-ef450 (1D3), Ly6G (1A8), CD4 (GK1.5), CD44 (IM7), CD62L (MEL-14) (Invitrogen, BioLegend, Tonbo Biosciences). Gp33-specific CD8 T cells were stained for 45 minutes at room temperature with H2D^b^/(KAVYNFTAC) tetramer (National Institutes of Health Tetramer Core Facility). For intracellular cytokine staining, splenocytes were stimulated *ex vivo* with LCMV gp33-41 peptide at a concentration of 0.4μg/L (AnaSpec) in the presence of Brefeldin A (Invitrogen) and GolgiStop (BD Biosciences) for 4 hours. Cells were then washed with FACS buffer, permeabilized and fixed using a fixation/permeabilization kit (BD Biosciences) per the manufacturer’s instruction. Cells were then stained with aIFNg (XMG1.2) and aTNF (MP6-XT22) antibodies for 1 hour at room temperature. For phospho-flow staining, cells were fixed with warmed BD Pharmingen™ Phosphoflow Fix Buffer I and permeabilized with BD Pharmingen™ Phosphoflow Fix Buffer III per manufacturer’s instructions. Cells were then washed and stained with phospho-STAT1 fluorescently labeled antibody (pY701) (BD Biosciences) for 1 hour at room temperature and washed. Samples were collected using a LSR II flow cytometer (BD Biosciences) and data was analyzed using FlowJo software (v10.8.0). For CD8 isolation for RNA sequencing, splenic CD8 T cells (CD19^−^TCRb^+^CD4^−^CD8^+^) were sorted using FACSAria III (BD Biosciences) (purity > 95%), spun, and lysed in RLT buffer (Qiagen), shredded using QIAshredder (Qiagen), and stored at -80°C until use.

### 2.7 RNA sequencing and bioinformatics analysis

CD8 T cells were sort purified from the spleens of mice on day 9 p.i. (3 mice per group). RNA was isolated using the RNeasy Micro Kit (Qiagen). RNA quality and quantity were assessed using an Agilent 2100 Bioanalyzer with the Eukaryote Total RNA Pico kit (Tecan). Single primer isothermal amplification (SPIA)-cDNA was created using Tecan Ovation RNA sequencing system V2 protocol. Purified cDNA was then sheared (target base pair size of 300 nucleotides) with Covaris LE220 focused ultra sonicator (Woburn) using a 96-microtube-50 AFA fiber plate. Libraries were created using KAPA Hyper-Prep kit (Roche). The indexes used were UDI DNA indexes (Illumina). R package voom-limma (15) was used for count normalization and differential gene expression analysis. *P-*value < 0.05 and Log (Fold Change)>1 was used to determine significance. Ranked gene lists were used to run Gene Set Enrichment Analysis (GSEA) (16).

### 2.8 Statistical analyses

Statistical analyses were performed using GraphPad Prism Software (version 9) following consultation with a senior biostatistician in the Department of Biostatistics at St. Jude. Outliers were removed with Grubb’s test using GraphPad Prism outlier calculator. Kruskal-Wallis test was performed followed by Mann-Whitney test to determine statistical significance. *P-*value <0.05 (*), *P-*value < 0.01 (**), *P-*value <0.001 (***), *P-*value <0.0001 (****).

## 3 Results

### 3.1 Ruxolitinib improves disease manifestations in *Prf1−/−* mice infected LCMV-WE

**Figure 1:**
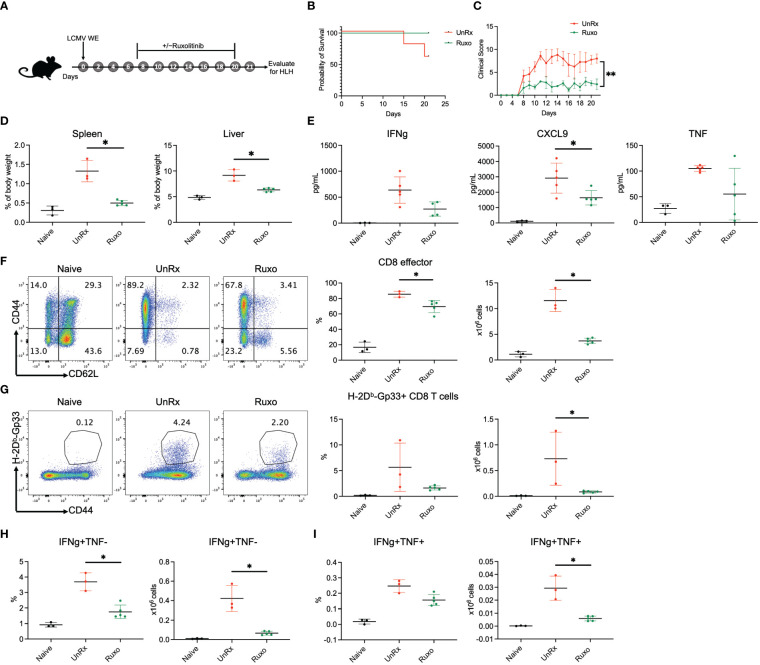
Ruxolitinib lessens the manifestations of disease in *Prf1*−/− mice infected with LCMV WE. **(A)** Schematic representation of the experiment; *Prf1*−/− mice were infected with LCMV WE and left untreated (UnRx) or treated with ruxolitinib (as described in the Methods) starting on day 7 p.i. and continuing until day 20 p.i. Naïve mice served as a negative control. Mice were euthanized and HLH parameters assessed on day 21 p.i. Survival curves **(B)** and clinical scores **(C)** of mice that were infected with LCMV WE and not treated (UnRX; 2 out of 5 mice died on day 15 and day 20 p.i.) or treated with ruxolitinib (5 of 5 mice survived). The clinical scores of mice that did not survive were excluded after the day of their euthanasia. **(D)** Spleen and liver weights depicted as a proportion of the final body weight. **(E)** Levels of serum IFNg, CXCL9 and TNF **(F)** Representative flow plots (left) and summary graphs (right) showing frequency and absolute numbers of splenic CD44+CD62L− effector CD8 T cells gated on total CD8 T cells. **(G)** Representative flow plots (left) and summary graphs (right) showing frequency and absolute numbers of splenic CD44+Gp33 tetramer+CD8 T cells. **(H)** Frequency (left) and absolute numbers (right) of IFNg+TNF− CD8 T cells gated on CD44+CD62L− effector CD8 T cells. **(I)** Frequency (left) and absolute numbers (right) of IFNg+TNF+ CD8 T cells gated on CD44+CD62L− effector CD8 T cells. Each data point represents one mouse. *P < 0.05.

A previous study has reported lack of efficacy and undue toxicity when treating LCMV WE-infected *Prf1*−/− mice with high doses of ruxolitinib (90 mg/kg twice daily) (10). Nevertheless, we have observed that this dose is effective and well-tolerated when treating LCMV Armstrong-infected animals (7, 8, 13). To explore this discrepancy, we infected *Prf1*−/− mice with LCMV WE and then treated animals with ruxolitinib at 90 mg/kg orally twice daily from day 7 until day 20 p.i. Mice were euthanized and HLH parameters evaluated on day 21 p.i. (**Figure 1A**). In the representative experiment shown, 100% (4/4) of ruxolitinib-treated *Prf1*−/− mice survived while only 60% (3/5) of untreated (UnRx) mice did so (**Figure 1B**). Ruxolitinib-treated mice also exhibited significantly lower clinical scores (indicating less severe disease; **Figure 1C**) and no signs of toxicity; none of the ruxolitinib-treated mice died and all of the treated mice had improved clinical scores compared to UnRx. Further, ruxolitinib significantly improved organomegaly (**Figure 1D**) and reduced the serum levels of the IFNg effector CXCL9 (**Figure 1E**). At this time point, serum IFNg levels were minimal to nil (**Figure 1E**). Ruxolitinib treatment also significantly reduced the frequency and/or absolute number of effector CD44+CD62L− CD8 T cells (**Figure 1F**), Gp33-specific CD8 T cells (**Figure 1G**), and the capacity of CD8 T cells to produce IFNg (**Figure 1H**), or both IFNg and TNF (**Figure 1I**) upon restimulation with gp33 peptide. Collectively, these findings demonstrate that high-dose ruxolitinib is well-tolerated and improves the survival, clinical features, and CD8 T cell number and cytokine production in *Prf1*−/− mice infected with LCMV WE.

### 3.2 Single agent or combination therapy differentially impact disease features in Prf1−/− mice infected with LCMV Armstrong

**Figure 2:**
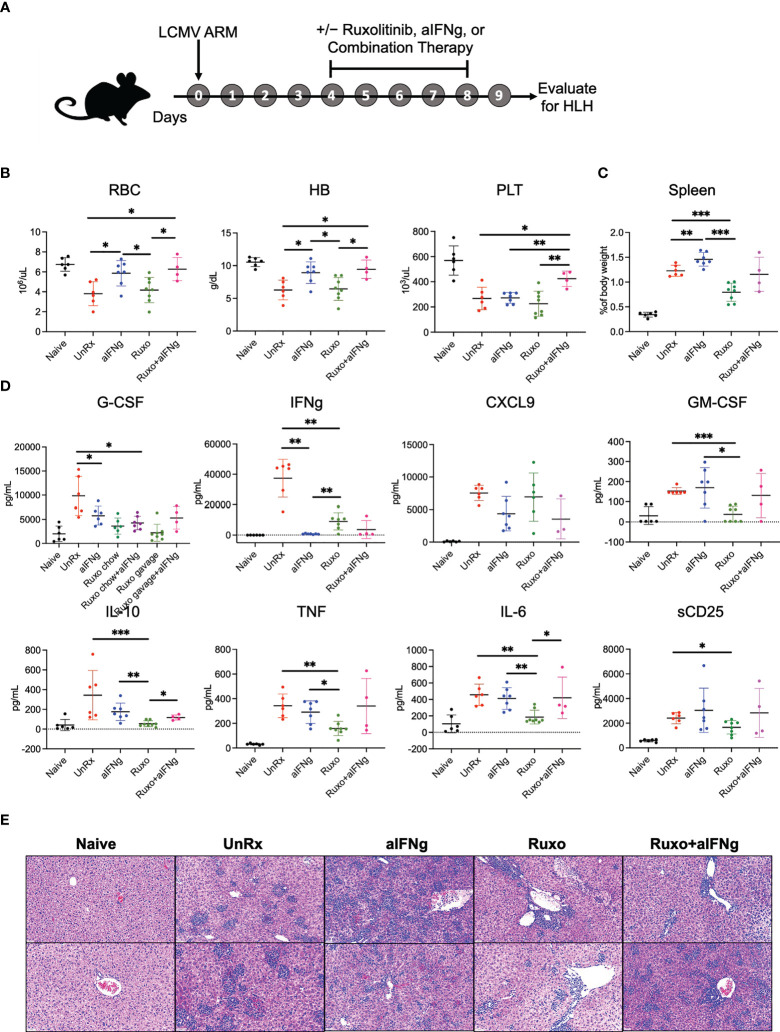
aIFNg, ruxolitinib, and combination treatment of *Prf1*−/− mice infected with LCMV Armstrong. **(A)** Schematic representation of the experiment; *Prf1*−/− mice infected with LCMV Armstrong (LCMV ARM) and treated with IFNg neutralizing antibody (aIFNg), ruxolitinib, or a combination of both agents (as described in the Methods) starting day 4 and continuing until day 8 p.i. Mice were then euthanized and multiple HLH parameters were evaluated on day 9 p.i. Naïve mice served as a negative control. **(B)** Number of red blood cells (RBC) (10^6^/μL), hemoglobin (HB) concentration (g/dL) and number of platelets (PLT) (10^3^/μL) in the various mouse cohorts. **(C)** Spleen weights depicted as a proportion of the final body weight. **(D)** Concentration of serum cytokines in the blood. Data points were pooled from two experiments and each data point represents one mouse. Outliers were removed using Grubb’s test **(E)** Representative images of hematoxylin and eosin-stained liver sections shown at a magnification of 20X; Each sample represents one liver section from one mouse. Sections from histological analysis were randomly chosen in a blinded fashion for inclusion in the Figure. *P < 0.05, **P < 0.01, ***P < 0.001.

As all of our prior studies utilized the LCMV Armstrong strain, we chose this model to further investigate the therapeutic effects of single agent aIFNg, single agent ruxolitinib, and combination therapy. Mice were treated with high dose aIFNg (500 μg every 3 days), high dose ruxolitinib (90mg/kg orally twice daily), or both of these agents (ruxo+aIFNg) starting on day 4 p.i. Mice were humanely euthanized on day 9 p.i. followed by evaluation for HLH disease parameters (**Figure 2A**). In this study, none of the treated animals died, regardless of whether single agent or combination therapy was used (***data not shown***). As we observed previously (7), aIFNg appeared most effective in preventing anemia (**Figure 2B**) and reducing serum IFNg levels (**Figure 2D**), and it did so when administered alone or along with ruxolitinib. In contrast, ruxolitinib was more effective in improving splenomegaly (**Figure 2C**), reducing hypercytokinemia (**Figure 2D**), and mitigating tissue inflammation (**Figure 2E**). Notably, for each of these latter disease manifestations, combination therapy was no better than either treatment alone – and at times, less effective than – treatment with ruxolitinib.

### 3.3 Single agent or combination therapy differentially impact myeloid and T cell numbers and activation status in *Prf1−/−* mice infected with LCMV Armstrong

**Figure 3:**
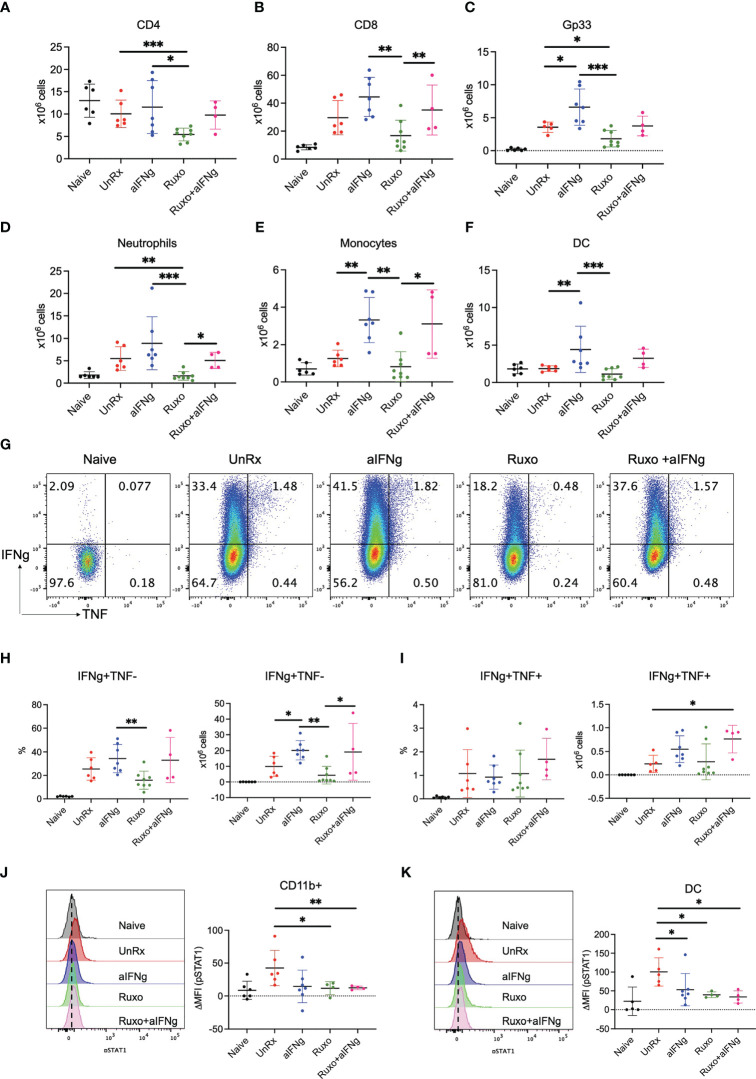
Impact of aIFNg, ruxolitinib, or combination treatment on immune cell numbers and function in *Prf1−/−* mice infected with LCMV Armstrong. On day 9 p.i., splenocytes from naïve mice, or LCMV-infected mice that were UnRx or treated with aIFNg, ruxolitinib, or combination treatment were analyzed by flow cytometry. Absolute numbers of splenic CD4 T cells **(A)**, CD8 T cells **(B)**, Gp33 tetramer-positive CD8 T cells **(C)**, neutrophils **(D)**, monocytes **(E)**, and dendritic cells (DCs; **F**). Representative dot plots **(G)** and summary graphs **(H, I)** showing the frequencies and absolute numbers of IFNg and/or TNF-producing effector CD8 T cells following ex vivo restimulation with gp33 peptide. Cells were gated on CD44+CD62L− effector CD8 T cells. Representative histograms (left) and summarized data (right) of the delta mean fluorescence intensity (MFI) of STAT1 phosphorylation in CD11ḅ myeloid cells in relation to MFI in naïve mice **(J)** and DCs **(K)**. Data points were combined from two independent experiments and each data point represents one mouse. *P < 0.05, **P < 0.01.

HLH is driven by the excessive accumulation and activation of T and myeloid cells. Therefore, we next examined how treatment with aIFNg, ruxolitinib, and combination therapy impacted T and myeloid cell numbers, functions or intracellular signaling. When compared to aIFNg, ruxolitinib was significantly more effective at reducing the absolute numbers of splenic CD4 and CD8 T cells, gp33-specific CD8 T cells, monocytes, neutrophils, and DCs (**Figures 3A–F**; see gating strategy in **Supplemental Figure 1**). Curiously, combination treatment was less effective than ruxolitinib in reducing cell numbers, particularly as relates to neutrophils, monocytes and DCs. Ruxolitinib also appeared more effective than aIFNg at reducing the proportions and numbers of IFNg-producing CD8 T cells (**Figures 3G, H**), with combination treatment reversing this effect. The proportions and numbers of IFNg and TNF co-producing CD8 T cells were comparable across all treatment groups (**Figure 3I**). Despite their differential impacts on cell number, flow cytometric analyses demonstrated that all three treatments comparably reduced the phosphorylation of STAT1, the major STAT protein functioning downstream of IFNg, in splenic myeloid cells and DCs (**Figures 3J, K**).

### 3.4 Impact of single agent or combination therapy on the transcriptional profiles of splenic CD8 T cells from *Prf1−/−* mice infected with LCMV Armstrong

**Figure 4:**
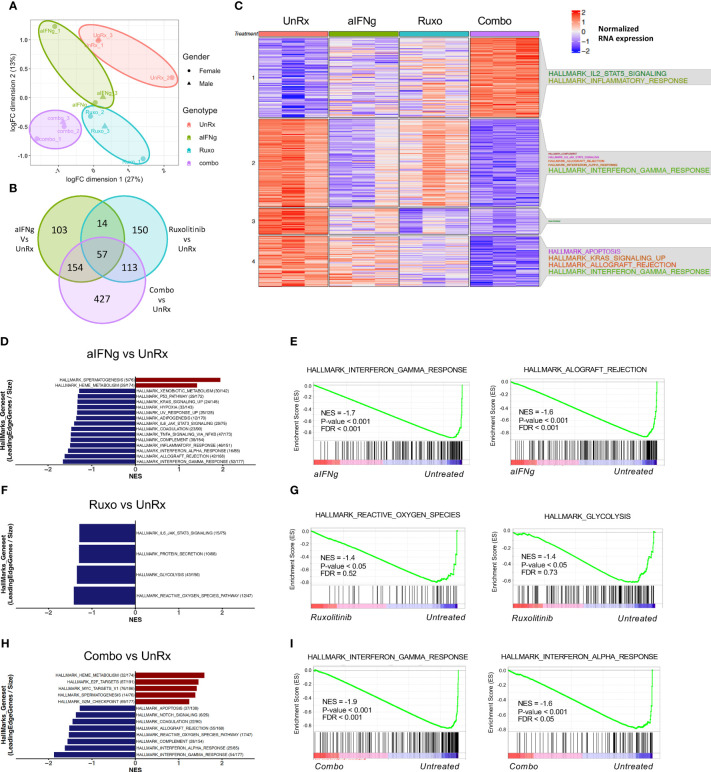
Changes in transcriptional profiles of splenic CD8 T cells from *Prf1*−/− mice infected with LCMV and treated with aIFNg, ruxolitinib, or combination treatment. **(A)** Unsupervised clustering using multidimensional scaling (MDS) of CD8 T cells infected with LCMV and left untreated (red) or treated with aIFNg (green), ruxolitinib (blue), or both agents (purple; 3 mice per group). **(B)** Venn diagram depicting the numbers of differentially expressed genes (DEGs) in aIFNg *vs.*UnRx, ruxolitinib *vs.* UnRx, combination vs. UnRx groups (*P-*value < 0.05, LogFC >1). **(C)** Heatmap of the significant DEGs in naïve, UnRx, aIFNg, ruxolitinib, and combination treatment groups annotated with enriched hallmark gene sets within each cluster. Bar graphs of GSEA demonstrating the most significant (P-value < 0.05 or P-value < 0.1 & normalized enriched score (NES) >1) hallmarks gene sets in CD8 T cells from LCMV-infected aIFNg-treated vs. UnRx mice **(D)**, LCMV-infected ruxolitinib-treated vs. UnRx mice **(F)**, or LCMV-infected combination treated vs. UnRx mice **(H)**. Enrichment plots highlighting the most significant pathways downregulated in CD8 T cells from LCMV-infected aIFNg-treated vs. UnRx mice **(E)**, LCMV-infected ruxolitinib-treated vs. UnRx mice **(G)**, or LCMV-infected combination treatment vs. UnRx mice **(I)**.

Understanding how specific therapies impact immune cell activation is crucial to our knowledge of treatment response and how to optimize therapy. To gain further insights, we performed RNA sequencing on CD8 T cells sorted on day 9 p.i. from the spleens of *Prf1*−/− mice that were infected or not with LCMV Armstrong and treated or not with aIFNg, ruxolitinib, or combination therapy (**Figure 4**). Following batch and sex correction, unsupervised clustering using multidimensional scaling (MDS) showed separation by treatment group (**Figure 4A**). Analysis of the most highly differentially expressed genes (DEGs) (P-value <0.05, LogFC >1) revealed 150 genes uniquely impacted by ruxolitinib treatment, 103 genes impacted by aIFNg treatment, and 427 genes impacted by combination treatment when compared to LCMV-infected but untreated (UnRx) cells (**Figure 4B**). To understand the biological processes enriched in each cluster, we performed over-representation analysis using hallmark gene sets to examine the global transcriptional profiles of CD8 T cells following various treatments compared to CD8 T cells from LCMV-infected but untreated mice (UnRx) (**Figure 4C**). As expected, in CD8 T cells from UnRx mice, there was upregulation of pathways involved in IFNg signaling, allograft rejection, KRAS signaling, and IL-6/JAK/STAT3 signaling (clusters 2 and 4). As expected, aIFNg and combination treatment nicely downregulated the expression of IFNg response genes (cluster 2), while ruxolitinib did so less effectively. Surprisingly, combined treatment with aIFNg and ruxolitinib, but not treatment with aIFNg or ruxolitinib alone, markedly induced the expression of IL-2, STAT5, and inflammatory response genes (cluster 1). Due to the lower number of genes, we could not determine the hallmarks of genes in cluster 3, which represents genes downregulated following ruxolitinib or combination treatment. Further analysis of this cluster revealed downregulation of genes associated with cell proliferation, such as, *Lrg1*, *Ngp*, *Olfml2b*, and *Lcn2* (17–20) and cell activation (e.g. *Mmp9*) (21) (**Supplemental Figure 2**).

To identify global changes in gene expression across multiple groups, we then performed gene-set enrichment analysis (GSEA) (16) using CD8 T cell data from naïve mice, or LCMV-infected mice that were UnRx, aIFNg-treated, ruxolitinib-treated, and aIFNg/ruxolitinib-treated. Comparison of gene expression in UnRx versus naïve CD8 T cells revealed a significant increase in G2M checkpoint, E2F targets, IFNg response, IL-2 STAT5 signaling, and IFNa response genes and a decrease in anti-inflammatory genes of the TGF-beta pathway (**Supplemental Figures 3A-C**), consistent with the CD8 T cell proliferation and effector activation in pHLH. We next sought to elucidate the main pathways targeted by aIFNg, ruxolitinib, or combination treatment, when compared to UnRx cells (**Figures 4D, E**). Anti-IFNg treatment was associated with reduced expression of IFNg response genes, and the allograft rejection, IFNa response, inflammatory response, and complement pathways, among others (**Figures 4D, E**). Analysis of the top 20 enriched genes in each of these pathways revealed downregulation of genes associated with CD8 T cell activation and effector function, including IFN regulator factor 8 (*Irf8*) (22), Granzyme A (*Gzma*), *Cd69*, *Icam1*, *Tnf*, and *Stat1;* as well as genes involved in memory CD8 T cell differentiation (e.g. *Il15ra*) (**Supplemental Figure 4A**). Of note, aIFNg treatment also led to the decreased expression of immunoregulatory genes, such as, *Il10ra* and *Il10*; and genes associated with the inhibition of IFNg production and CD8 T cell activation, such as, *Tnfaip3* (the tumor necrosis factor alpha-induced protein 3 (23) and *Ncr1* (natural cytotoxicity receptor-1) (24) (**Supplemental Figure 4A**). Interestingly, analysis of the gene set with hallmark ‘heme metabolism’ demonstrated an increase in the expression of genes associated with inflammasome activation due to upregulated toll-like receptor signaling such as, *Nek7* (NIMA related kinase 7) (25) and the ubiquitin ligase *Rnf19a* (26) (**Supplemental Figure 4B**). Therefore, while IFNg neutralization effectively lessened expression of genes required for CD8 activation and effector function, it also decreased the expression of genes with inhibitory functions in T cells.

GSEA analysis of genes affected by ruxolitinib treatment compared to UnRx demonstrated fewer significantly impacted pathways with decreased expression of genes related to reactive oxygen species, glycolysis, protein secretion, and IL-6/JAK/STAT3 signaling (**Figures 4F–G**). Among the downregulated genes included super oxide dismutase 2 (*Sod2*), which is associated with redox regulation in CD8 T cells (27) and glutamate cysteine ligase catalytic subunit (*Gclc*), which is activated in CD8 T cells upon T cell-receptor (TCR) engagement and is essential in mouse T cells to enable MYC-dependent metabolic reprogramming allowing for activated T cells to switch to glycolysis (28). (**Supplemental Figure 4C**). Ruxolitinib also reduced expression of genes involved in protein secretion, including Cathepsin C (*Ctsc*) (29), and lysosome function (e.g. Galactosidase alpha (*Gla*), Palmitoyl-protein thioesterase 1 (*Ppt1*)) (**Supplemental Figure 4D**). Finally, ruxolitinib targeted multiple genes in the IL-6/JAK/STAT3 pathway, in line with the known role of JAK1 and JAK2 in mediating IL-6 signaling (30–33) (**Supplemental Figure 4D**). Altogether, ruxolitinib treatment reduced expression of pathways and genes critical for CD8 T cell activation and energy utilization.

We next examined the main pathways targeted by combination therapy. Compared to CD8 T cells from UnRx mice, cells exposed to aIFNg and ruxolitinib exhibited reduced expression of IFNg and IFNa response and complement pathway genes (**Figures 4H, I**). This decrease is likely driven by aIFNg as these genes and pathways largely overlapped with those downregulated in CD8 T cells from mice treated with single agent IFNg (**Figures 4D, E**). Similarly, both aIFNg and combination therapy led to upregulation of genes involved in heme metabolism again suggesting attribution to aIFNg treatment. Unexpectedly, combination treatment led to increased expression of genes in the E2F and MYC pathways indicative of cell proliferation (**Figures 4H, I**). Among the upregulated genes were *Prps1* (Phosphorybosyl Pyrophosphate Synthetase 1), *Hells* (Helicase, lymphoid specific), *Cse1l* (Chromosome segregation 1 like), *Dck* (Deoxycytidine kinase), *Slbp* (Stem-loop binding protein), and *Nap1l1* (Nucleosome assembly protein 1 like 1) (**Supplemental Figure 4D**) (34–39).

**Figure 5:**
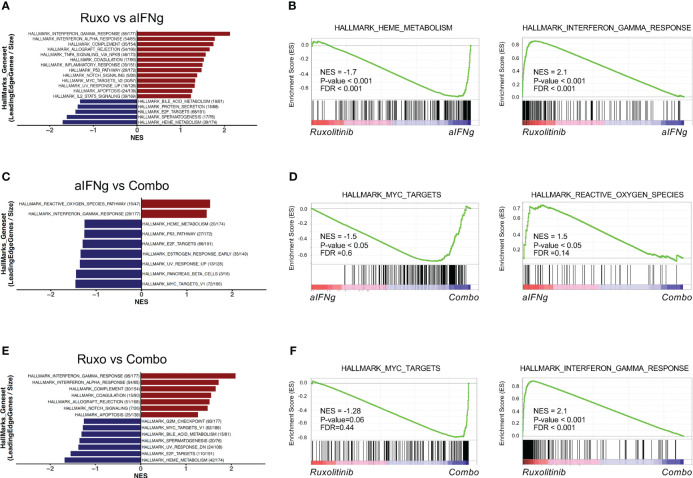
Top hallmark pathways in splenic CD8 T cells from *Prf1*−/− mice infected with LCMV and treated with aIFNg, ruxolitinib, or combination treatment. Bar graphs of GSEA demonstrating the most significant (P-value < 0.05 or P-value < 0.1 & NES >1) hallmark gene sets in CD8 T cells from LCMV-infected mice treated with ruxolitinib vs. aIFNg **(A)**, aIFNg vs. combination therapy **(C)**, or ruxolitinib vs. combination therapy **(E)**. Enrichment plots highlighting the most significant pathways downregulated or upregulated with ruxolitinib vs. aIFNg **(B)**, aIFNg vs. combination therapy **(D)**, or ruxolitinib vs. combination therapy **(F)**.

Finally, we sought to differentiate the main pathways targeted between the three treatment groups. Similar to our prior analysis, aIFNg appeared to effectively target pathways involving the IFNg response, IFNa response and complement genes, while ruxolitinib better downregulated genes involved in pathways involving heme metabolism, E2F targets, and protein secretion (**Figures 5A, B**; **Supplemental Figures 5A, B**). Comparison of aIFNg and ruxolitinib to combination treatment revealed that IFNg response genes were more effectively targeted by combination treatment (**Figures 5C–F**; **Supplemental Figures 5C–F**), while genes related to cell proliferation were less effectively targeted (**Figures 5C–F**; **Supplemental Figures 5C-F**). Together, these data demonstrate that aIFNg, ruxolitinib and combination treatment exert their effects through shared as well as distinct pathways which differentially impact T cell proliferation, activation, and energy metabolism.

## 4 Discussion

Knowledge of pHLH pathophysiology has expanded tremendously in recent years through the use of mouse models, which have revealed that excessive production of pro-inflammatory cytokines by overactive cells of the immune system drives the signs and symptoms of disease. Accordingly, targeting these cytokines has become a major therapeutic focus with the IFNg-neutralizing antibody emapalumab and the JAK1/2 inhibitor ruxolitinib demonstrating efficacy in mouse pre-clinical models and humans with pHLH. Herein, we show that ruxolitinib is well-tolerated and lessens disease features following infection of *Prf1*−/− mice with two different viral strains, LCMV Armstrong and LCMV WE. Since pHLH can be triggered by a variety of pathogens that induce differing patterns of cytokine production, these findings suggest that ruxolitinib will be effective when HLH is induced across a spectrum of infectious agents. In line with this possibility, ruxolitinib has demonstrated benefit in treating HLH in patients with Epstein-Barr virus (40, 41), cytomegalovirus (42), influenza (43), histoplasmosis (44, 45), malaria (46), and disseminated tuberculosis infections (47).

We also demonstrate that the combination of high doses of ruxolitinib and aIFNg is not toxic when administered to LCMV-infected *Prf1*−/− mice; however, this regimen is not necessarily more effective than treatment with either drug alone. Indeed, at times combination treatment appeared to reverse the beneficial effects of ruxolitinib, particularly as relates to reducing myeloid, DC, and CD8 T cell expansion and T cell cytokine production. These findings are suggestive of an immunoregulatory role for IFNg. Indeed, it has been widely demonstrated that IFNg is an important cytokine during the contraction phase of the T cell response following viral (48–50) and mycobacterial infections by promoting T cell apoptosis (51). Consistent with these observations, the apoptotic program is triggered in IFNg-stimulated T cells *via* the activation of STAT1 and IRF-1 (52). In contrast, IFNg-deficient CD4 T cells are rendered resistant to activation-induced cell death (AICD) (51, 53). Other studies using IFNg receptor knockout mice demonstrate that upon LCMV infection, T cells are hyperproliferative and less susceptible to AICD (54) and antigen-specific CD8 T cells persist in higher numbers in *Prf1*−/− *Ifng*−/− mice infected with an attenuated strain of *Listeria monocytogenes* (50). This regulatory role of IFNg also expands to myeloid cells, with LCMV-infected IFNg-deficient *Prf1*−/− mice developing severe hyperinflammation with evidence of neutrophilia and an altered cytokine milieu dominated by IL-6, IL-1β, and GM-CSF (55). Altogether, these studies demonstrate that in addition to its activating properties, IFNg plays important immunoregulatory roles, with complete blockade or genetic ablation exacerbating immune responses following various infections. Therefore, it is possible that greater abrogation of IFNg signaling through the combined use of high doses of ruxolitinib and aIFNg (as was done in this study) might be counterproductive in suppressing inflammation in pHLH.

From a clinical perspective, glucocorticoids, which have pro-apoptotic properties, are often used to treat HLH. However, elevated cytokines, such as, IL-2, 7, and 15, which signal through the JAK-STAT pathway, confer resistance of CD8 T cells to dexamethasone-induced cell death (8). Indeed, we previously showed that incubation of activated mouse CD8 T cells with ruxolitinib (to block JAK-STAT signaling) restores T cell apoptotic potential following dexamethasone exposure despite the presence of excess exogenous IL-2 (8). Therefore, if dexamethasone was given along with ruxolitinib and an anti-IFNg neutralizing antibody, it is possible that the proliferative and anti-apoptotic effects of complete IFNg neutralization could be overcome.

Our findings differ from those of Joly et al., who reported that combination therapy with ruxolitinib and aIFNg was more effective than treatment with ruxolitinib alone (11). However, the dose of ruxolitinib used in the study by Joly was much lower than the one used in our study (4 mg/kg vs. 90 mg/kg, respectively). Consistent with the lower dosing in the study by Joly, ruxolitinib failed or only marginally improved peripheral blood cytopenias, serum ferritin and cytokine levels, and T cell STAT phosphorylation. Thus, the benefit of combination therapy appears to have been largely driven by aIFNg. The aIFNg dose used by Joly was also lower than ours (200 μg vs. 500 μg, respectively), which may have served to reduce the likelihood of untoward effects in their study. The differences in outcome between these two studies suggest that dose titration will be important to maximize the beneficial and lessen the adverse effects of combination therapy. In support of this notion is a recent case report describing a patient with refractory EBV-HLH who was treated with emapalumab followed by increasing doses of ruxolitinib with subsequent control of disease (56).

Since CD8 T cells are central to driving pHLH, we sought to elucidate the transcriptional landscape of these cells in LCMV-infected *Prf1*−/− mice that had or had not been treated with aIFNg, ruxolitinib, or both of these agents. Analysis of the global transcriptional landscape revealed a larger set of genes targeted by aIFNg and combination therapy when compared to ruxolitinib alone. In this investigation, mice received the last dose of ruxolitinib on day 8, the evening prior to CD8 T cell and RNA isolation. Therefore, the impacts of ruxolitinib treatment may have been less pronounced due to the short half-life of the drug. Nevertheless, we observed that ruxolitinib significantly decreased the expression of genes involved in pathways important for CD8 T cell activation and energy metabolism, such as reactive oxygen species, protein secretion, and glycolysis. In contrast, targeting IFNg, alone or in combination with ruxolitinib, was more effective in inhibiting pathways downstream IFNg itself. Perhaps, one of the most surprising findings was the observation that combination therapy led to increased CD8 T numbers, T cell IFNg production, and expression of genes involved in cell survival (IL-2/STAT5 signaling) and proliferation (E2F and MYC pathways), when compared to cells treated with aIFNg or ruxolitinib monotherapy. These observations provide further evidence that complete inhibition of IFNg signaling may be detrimental in suppressing inflammation and that caution should be taken when combining aIFNg and ruxolitinib, especially when larger doses of these medications are employed.

## Data availability statement

All raw and processed RNAseq data associated with this manuscript are deposited to Gene Expression Omnibus (GEO), with the accession number GSE218500.

## Ethics statement

All experimental protocols were approved by the Institutional Animal Care and Use Committee and the Institutional Biosafety Committee.

## Author contributions

SA designed, performed the experiments, interpreted the data, and wrote this manuscript. NO performed RNA sequencing analysis. HT performed histological analyses of liver sections. AS, CK, and MB provided technical assistance. KN oversaw the project, interpreted the data, and edited the manuscript. All authors contributed to the article and approved the submitted version.

## Supplementary material

Supplementary Figure 1 Gating strategy to identify various leukocyte subsets in the spleen. The following leukocyte subsets were defined as follows: CD8 T cells (CD19−NK1.1−TCRb+CD8+); CD4 T cells (CD19−NK1.1−TCRb+CD4+); dendritic cells (DC) (CD19−NK1.1−TCRb−CD11c+); Monocytes (CD19−NK1.1−TCRb−CD11c−CD11b+Ly6C+Ly6G−); Neutrophils (CD19−NK1.1−TCRb−CD11c−CD11b+Ly6C+Ly6G+).

Supplementary Figure 2 Heatmap of genes of cluster 3. Heatmap demonstrating the name and expression levels of genes in cluster 3.

Supplementary Figure 3 Gene enrichment analysis (GSEA) in CD8 T cells UnRx *vs.* Naïve. **(A)** Bar graphs of GSEA demonstrating the most significant (P-value < 0.05 or P-value < 0.1 & NES >1) hallmark gene sets in CD8 T cells from LCMV-infected mice *vs.* naïve mice; in red hallmark gene set upregulated in UnRx compared to Naïve and in blue hallmark gene set downregulated in UnRx compared to naïve **(B)** Enrichment plots of representative hallmark gene sets **(C)** List of genes comprising the main hallmark gene sets that changed in UnRx compared to naïve CD8 T cells (G2M checkpoint, E2F targets, interferon gamma response, IL2/STAT5 signaling, TGF beta signaling).

Supplementary Figure 4 Leading edge genes in the top pathways identified by GSEA analysis. Heatmaps demonstrating the top 20 leading edge genes enriched in hallmark gene sets when comparing aIFNg to UnRx **(A, B)**, ruxolitinib to UnRx **(C, D)** and combination treatment to UnRx **(E, F)**.

Supplementary Figure 5 Leading edge genes in the top pathways identified by GSEA analysis. Heatmaps demonstrating the top 20 leading edge genes in enriched in hallmark gene sets when comparing ruxolitinib to aIFNg **(A, B)**, aIFNg to combination treatment **(C, D)**, and ruxolitinib to combination treatment **(E, F)**.
