# Supplementary figures and images for: Cellular and transcriptional impacts of Janus kinase and/or IFN-gamma inhibition in a mouse model of primary hemophagocytic lymphohistiocytosis
Source: Front Immunol. 2023 Apr 27;14:1137037. doi: 10.3389/fimmu.2023.1137037 (PMC10204641; doi:10.3389/fimmu.2023.1137037)

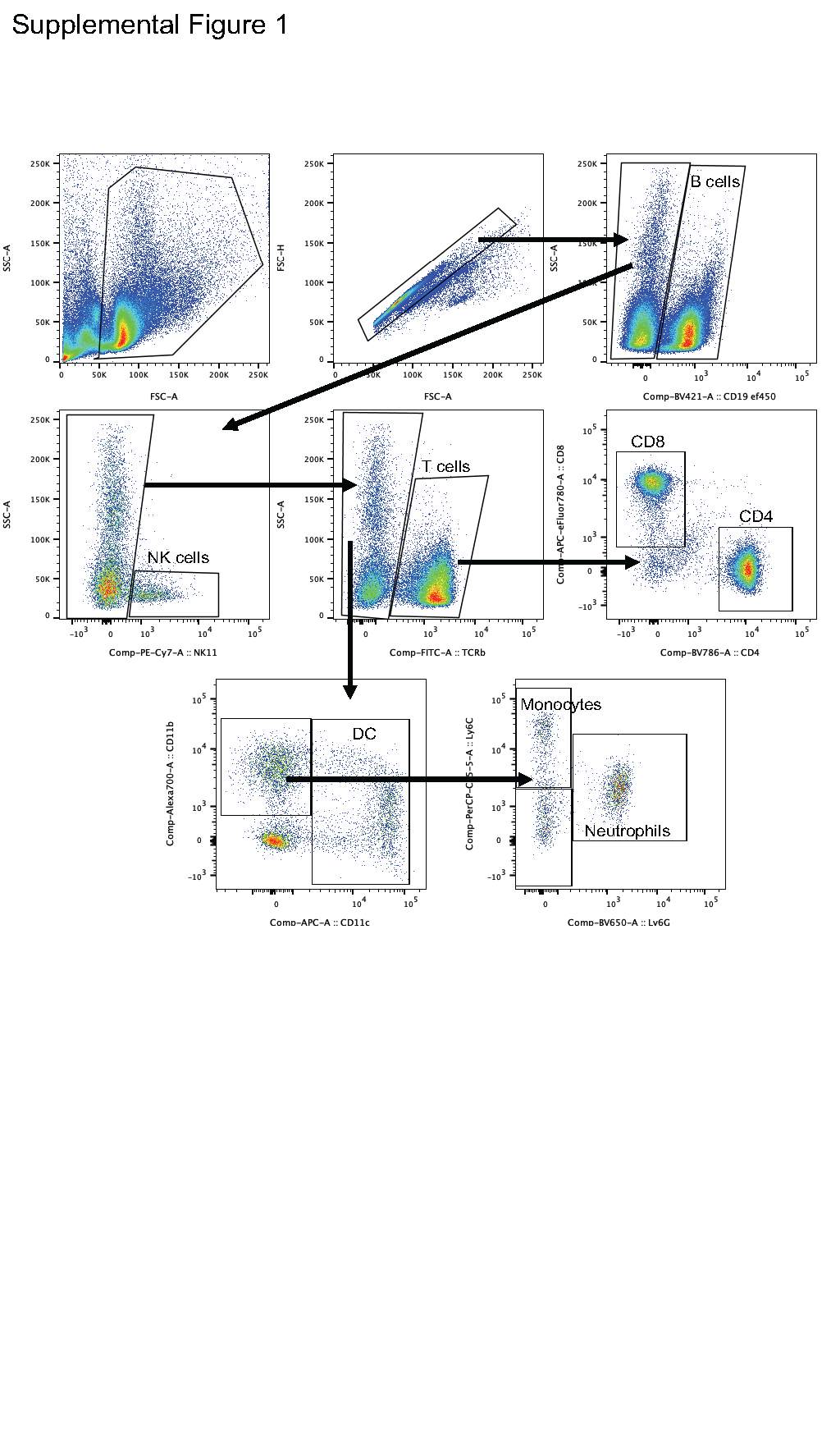

Supplement: Supplementary Figure 1 — Gating strategy to identify various leukocyte subsets in the spleen. The following leukocyte subsets were defined as follows: CD8 T cells (CD19−NK1.1−TCRb+CD8+); CD4 T cells (CD19−NK1.1−TCRb+CD4+); dendritic cells (DC) (CD19−NK1.1−TCRb−CD11c+); Monocytes (CD19−NK1.1−TCRb−CD11c−CD11b+Ly6C+Ly6G−); Neutrophils (CD19−NK1.1−TCRb−CD11c−CD11b+Ly6C+Ly6G+). [file Image_1.tiff]

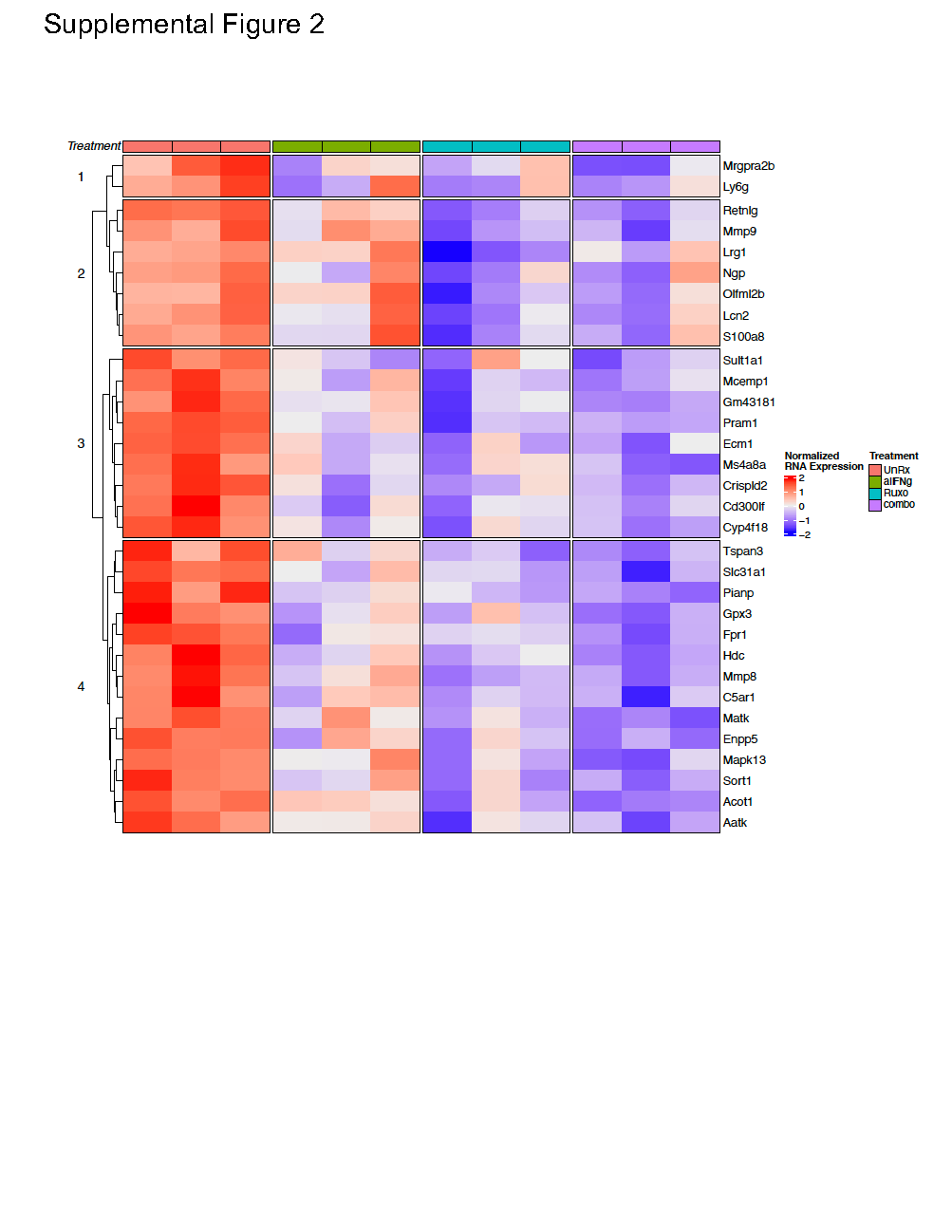

Supplement: Supplementary Figure 2 — Heatmap of genes of cluster 3. Heatmap demonstrating the name and expression levels of genes in cluster 3. [file Image_2.tiff]

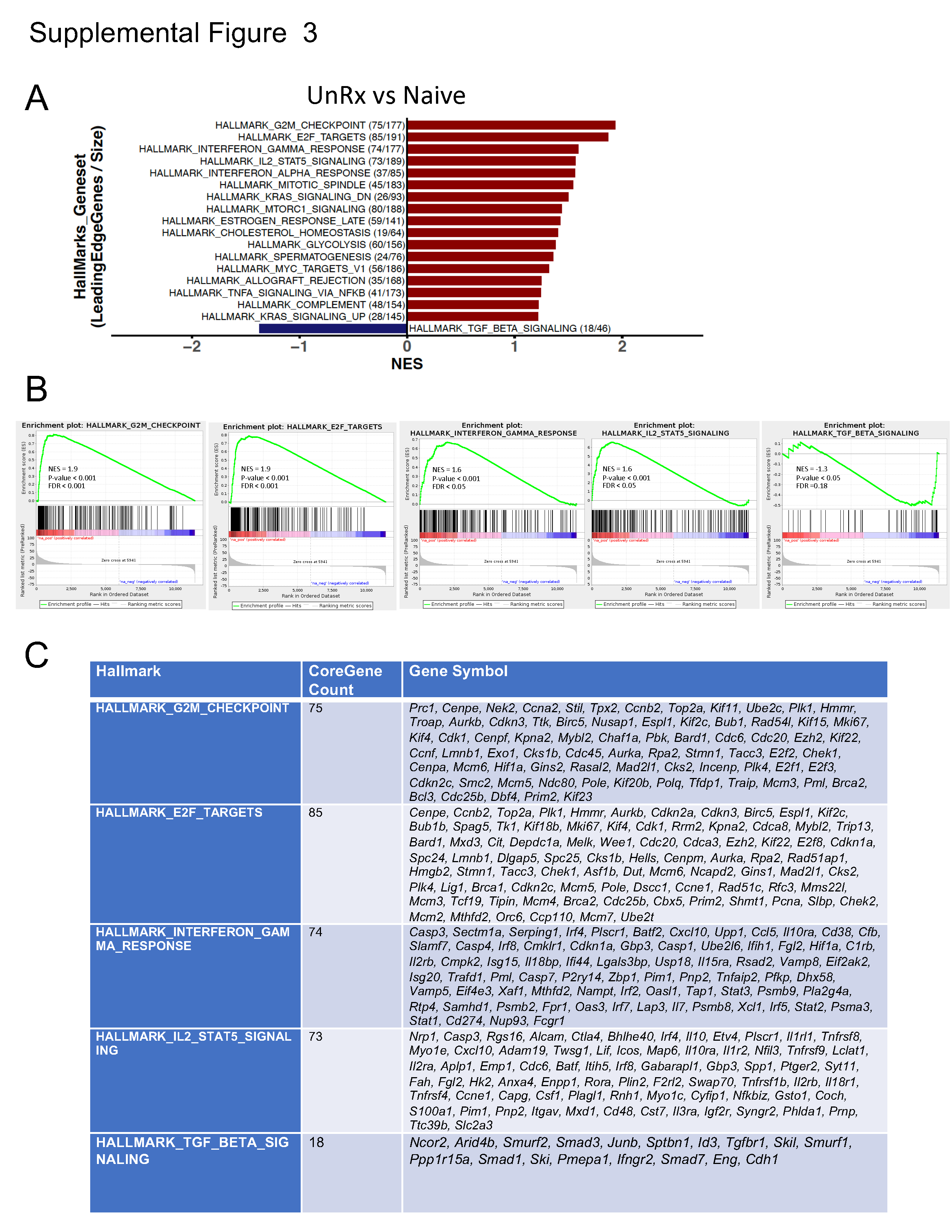

Supplement: Supplementary Figure 3 — Gene enrichment analysis (GSEA) in CD8 T cells UnRx vs. Naïve. (A) Bar graphs of GSEA demonstrating the most significant (P-value < 0.05 or P-value < 0.1 & NES >1) hallmark gene sets in CD8 T cells from LCMV-infected mice vs. naïve mice; in red hallmark gene set upregulated in UnRx compared to Naïve and in blue hallmark gene set downregulated in UnRx compared to naïve (B) Enrichment plots of representative hallmark gene sets (C) List of genes comprising the main hallmark gene sets that changed in UnRx compared to naïve CD8 T cells (G2M checkpoint, E2F targets, interferon gamma response, IL2/STAT5 signaling, TGF beta signaling). [file Image_3.tiff]

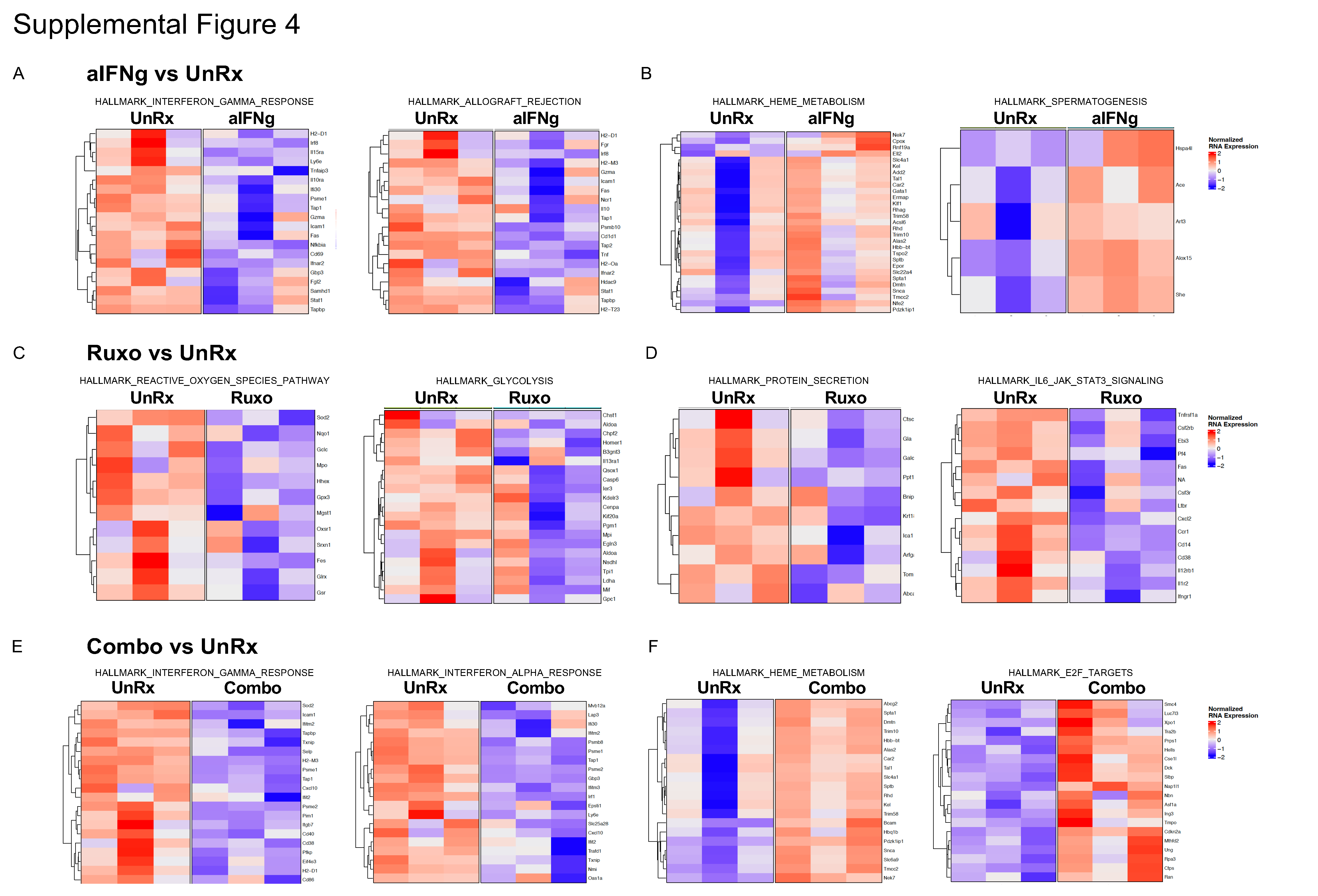

Supplement: Supplementary Figure 4 — Leading edge genes in the top pathways identified by GSEA analysis. Heatmaps demonstrating the top 20 leading edge genes enriched in hallmark gene sets when comparing aIFNg to UnRx (A, B), ruxolitinib to UnRx (C, D) and combination treatment to UnRx (E, F). [file Image_4.tiff]

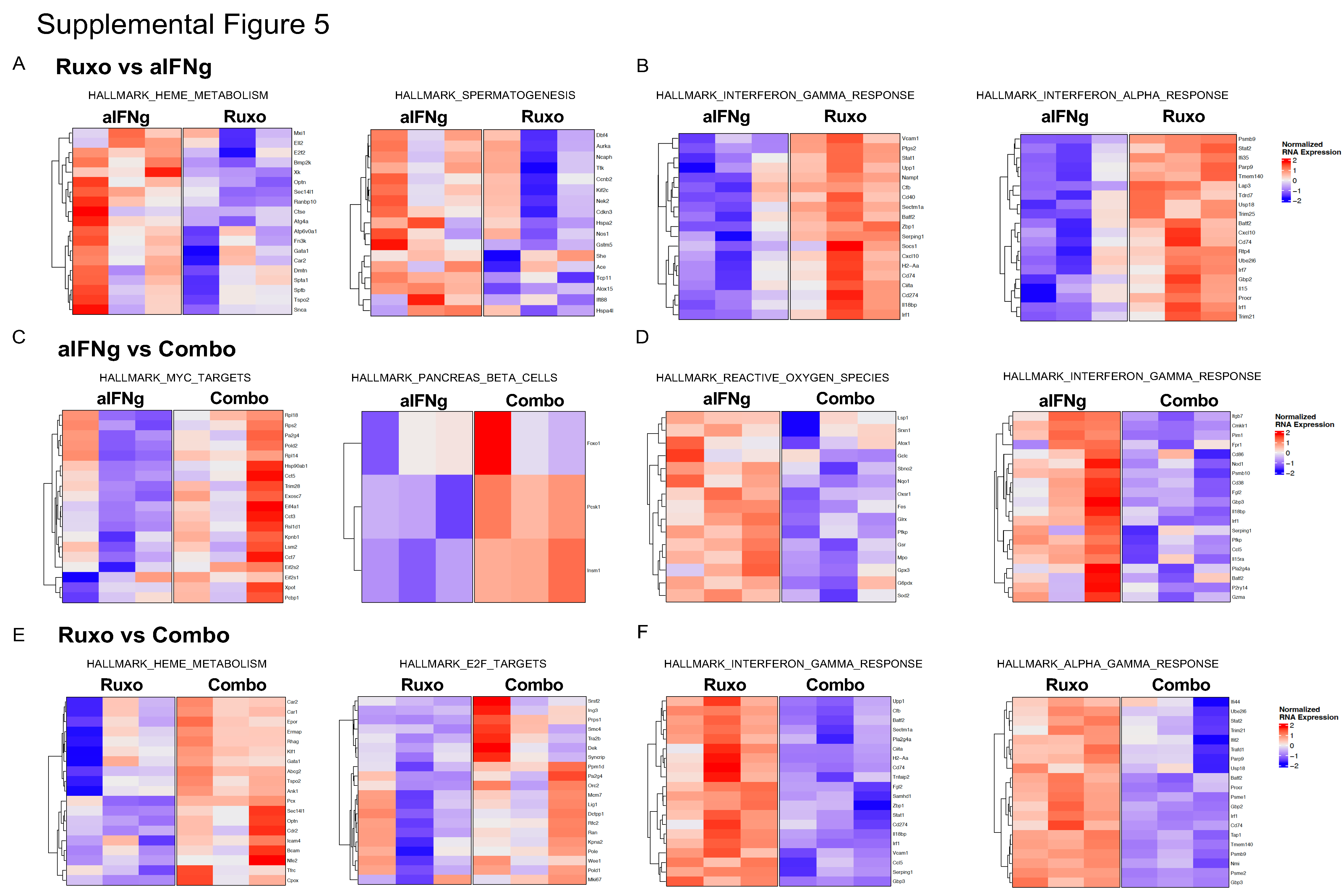

Supplement: Supplementary Figure 5 — Leading edge genes in the top pathways identified by GSEA analysis. Heatmaps demonstrating the top 20 leading edge genes in enriched in hallmark gene sets when comparing ruxolitinib to aIFNg (A, B), aIFNg to combination treatment (C, D), and ruxolitinib to combination treatment (E, F). [file Image_5.tiff]
